# Ethylene and plant responses to phosphate deficiency

**DOI:** 10.3389/fpls.2015.00796

**Published:** 2015-09-29

**Authors:** Li Song, Dong Liu

**Affiliations:** Ministry of Education Key Laboratory of Bioinformatics, Center for Plant Biology, School of Life Sciences, Tsinghua University, BeijingChina

**Keywords:** ethylene, phosphate responses, root architecture, transcriptional regulation, signaling, crosstalk

## Abstract

Phosphorus is an essential macronutrient for plant growth and development. Phosphate (Pi), the major form of phosphorus that plants take up through roots, however, is limited in most soils. To cope with Pi deficiency, plants activate an array of adaptive responses to reprioritize internal Pi use and enhance external Pi acquisition. These responses are modulated by sophisticated regulatory networks through both local and systemic signaling, but the signaling mechanisms are poorly understood. Early studies suggested that the phytohormone ethylene plays a key role in Pi deficiency-induced remodeling of root system architecture. Recently, ethylene was also shown to be involved in the regulation of other signature responses of plants to Pi deficiency. In this article, we review how researchers have used pharmacological and genetic approaches to dissect the roles of ethylene in regulating Pi deficiency-induced developmental and physiological changes. The interactions between ethylene and other signaling molecules, such as sucrose, auxin, and microRNA399, in the control of plant Pi responses are also examined. Finally, we provide a perspective for the future research in this field.

## Introduction

Plants are sessile organisms that acquire essential mineral nutrients from soils through their roots. Plants often encounter nutrient deficiency in natural ecosystems and in agricultural lands. P (Phosphorus) is an essential macronutrient for plant growth, development, and metabolism. Inorganic phosphate, the major form of P that plants assimilate, however, is highly immobile in most soils because it is converted to organophosphates by microorganisms or is fixed with metals (Bieleski, 1973). Although P is abundant in most soils, the availability of Pi for plant uptake is quite low (Raghothama, 2000). Pi deficiency has become one of the most important constraints on agricultural productivity.

**FIGURE 1:**
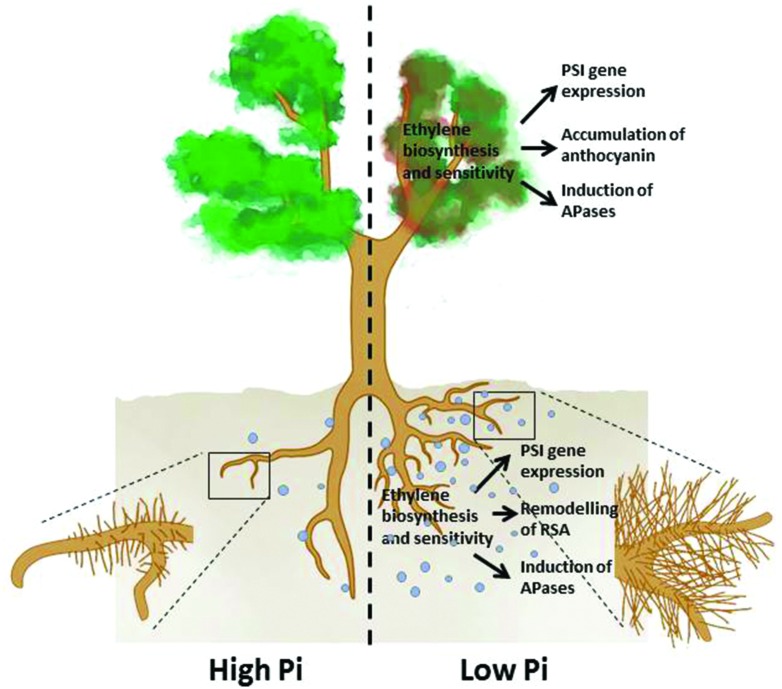
**An overview of the plant responses to Pi deficiency that involve ethylene.** Under Pi deficiency, primary root growth is inhibited, production of lateral roots and root hairs is enhanced (remodeling of RSA) APases are induced, expression of PSI genes is increased in both roots and shoots, and shoots accumulate more anthocyanins. The light blue dots denote root exudates, including APases, RNases, organic acids, and protons.

To cope with Pi deficiency, plants have evolved elaborate strategies to enhance acquisition and utilization of Pi from the environment and to conserve and reprioritize the internal use of Pi through recycling and redistribution processes. The major plant responses to Pi deficiency include: the active remodeling of RSA; the reduction in photosynthesis; the enhancement of high-affinity Pi transporter activities; the induction and secretion of APases, ribonucleases, and organic acids; the replacement of phospholipids in membranes with glycolipids and sulfolipids; and the accumulation of anthocyanin and starch (Vance et al., 2003; Yuan and Liu, 2008; **Figure 1**). These responses are modulated by sophisticated regulatory networks through both local and systemic signaling in which phytohormones play important roles (Chiou and Lin, 2011).

Ethylene, which is one of five classic phytohormones, regulates multiple aspects of plant development, such as seed germination, root growth, leaf abscission and senescence, and fruit ripening, as well as plant responses to biotic and abiotic stresses (Abeles et al., 1992). Ethylene was previously shown to be involved in Pi deficiency-induced remodeling of RSA. Recent research indicates that in addition to being a regulator of root growth, ethylene also participates in other plant responses to Pi deficiency (Nagarajan and Smith, 2012; Roldan et al., 2013). In this article, we will review the current knowledge about the roles of ethylene in plant responses to Pi deficiency. We will also provide a perspective about how research might increase our understanding of the molecular mechanisms by which ethylene regulates plant Pi responses.

## Ethylene Biosynthetic and Signaling Pathways

**FIGURE 2:**
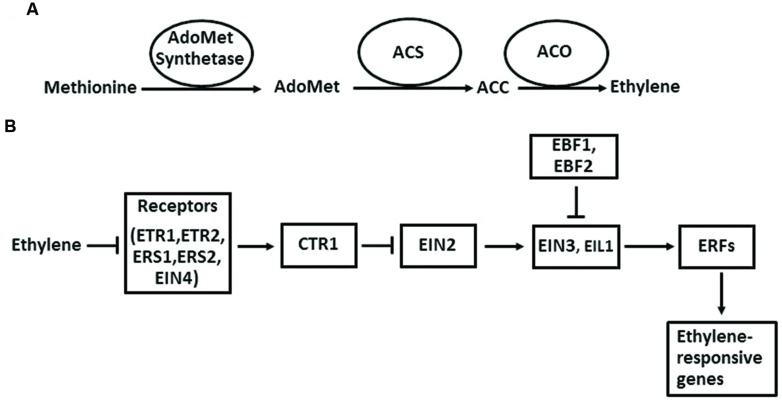
**The ethylene biosynthesis pathway in plants in general **(A)** and the ethylene signaling pathway in *Arabidopsis* (B).** Arrows indicate promotion, and perpendicular lines indicate inhibition.

The ethylene biosynthetic pathway has been well described in higher plants (Kende, 1993; Xu and Zhang, 2015; **Figure 2A**). Ethylene is produced from methionine, which is first converted to AdoMet by AdoMet synthetase. The next two steps are the conversion of AdoMet to ACC and the oxidative cleavage of ACC to form ethylene. The enzymes that catalyze these two reactions are ACS and ACO. Once ACC is formed in plant cells, it is automatically converted to ethylene by ACO in the presence of oxygen. The regulation of ethylene biosynthesis can be achieved by altering gene expression, protein stability, and enzymatic activity of ACS and ACO (McKeon et al., 1995; Bleecker and Kende, 2000).

The ethylene signaling pathway in *Arabidopsis* has provided a framework for the action mechanism of this hormone in higher plants (Zhao and Guo, 2011; Wen et al., 2015). In *Arabidopsis*, ethylene is perceived by its receptors, which are ETR1, ETR2, ERS1, ERS2, and EIN4 located in ER membrane. When ethylene levels are low, these five receptors activate the downstream component CTR1 through direct physical interaction. CTR1 belongs to the Raf-1 family of Ser/Thr protein kinases. The activated CTR1 suppresses the function of its target EIN2, an ER membrane-localized protein. When ethylene binds to the receptors, it disrupts the interaction between the receptors and CTR1, which inactivates CTR1. Therefore, the suppression of EIN2 by CTR1 is released. Next, a EIN2-C′ (C-terminal fragment of EIN2) is translocated to the nucleus. In the nucleus, EIN2-C′ enhances the levels of EIN3 and EIL1, two key transcription factors of the ethylene signaling pathway. EIN3 and EIL1 further activate the transcription of downstream target genes, such as ERFs, CHIB, PORA, and FLS2. Finally, the activation of these genes initiates a diverse array of plant responses to internal and external signals. In the absence of ethylene, EIN3 and EIL1 are degraded by the F-box proteins EBF1 (EIN3 BINDING F-BOX PROTEIN 1) and EBF2 through a 26S proteasome-mediated degradation pathway.

## Ethylene’s Role in Plant Responses to Pi Deficiency

### Pi Deficiency Alters Ethylene Biosynthesis

It has long been observed that Pi deficiency alters ethylene biosynthesis in plants. Some reports indicated that ethylene production was decreased in Pi-starved maize and tomato (Drew et al., 1989; Kim et al., 2008). In contrast, the increase of ethylene production was found in the roots of common bean under Pi deficiency (*Phaseolus vulgaris*), white lupin (*Lupinus albus*), and *Medicago falcata* (Borch et al., 1999; Gilbert et al., 2000; Li et al., 2009). Using quantitative reverse-transcription PCR (RT-qPCR), Lei et al. (2011b) showed that the expression of three members of the *ACS* gene family, *ACS2*, *ACS4*, and *ACS6*, was enhanced in *Arabidopsis* seedlings grown on a Pi-deficient medium. The enhanced expression of ethylene biosynthetic genes, mainly *ACS* and *ACO*, in Pi-starved *Arabidopsis* plants has also been demonstrated in several microarray and RNA-seq analyses (Misson et al., 2005; Morcuende et al., 2007; Calderon-Vazquez et al., 2008; Thibaud et al., 2010; Chacón-López et al., 2011; O’Rourke et al., 2013; Kang et al., 2014; Wang et al., 2014b). Morcuende et al. (2007) further found that the enhanced expression of *ACS2* and *ACS6* genes was reversed when Pi-deficient plants were resupplied with an adequate amount of Pi, indicating a causal relationship between the expression of these genes and the levels of Pi in the environment. The up-regulation of *ACO* genes by Pi deficiency was also detected in a variety of plant species, including common bean (Graham et al., 2006; Hernández et al., 2007), white lupin (Uhde-Stone et al., 2003; Wang et al., 2014b), and white clover (*Trifolium repens*; Roldan et al., 2013). In the plants examined, not all members of *ACS* and *ACO* families respond to Pi deficiency in the same manner. Thus, it is important to know how the transcription of different members of *ACS* and *ACO* gene families is affected by Pi deficiency in specific tissues and at specific developmental stages. Such information will increase our understanding of how ethylene biosynthesis is regulated in a spatiotemporal manner by Pi deficiency.

In *Arabidopsis*, PHR1 is a MYB-type transcription factor that binds to a *cis*-element with the imperfect palindromic sequence GNATATNC, a sequence that is prevalent in the promoters of PSI (Pi starvation-induced) genes (Rubio et al., 2001). PHR1 is regarded as a central regulator for transcriptional responses of plants to Pi starvation (Bustos et al., 2010). In *Arabidopsis*, PHL1 is a close relative of PHR1. In the *phr1phl1* double mutant, the induction of most PSI genes, including *ACS6* and *ACS7*, is impaired to different extents (Bustos et al., 2010). These results indicate that the increased expression of these two *ACS* genes is modulated by the central regulatory pathway of Pi responses. Interestingly, the expression of another two PSI *ACS* genes, *ACS2* and *ACS4*, is independent of the PHR1 pathway (Bustos et al., 2010), suggesting that the regulation of different members of the *ACS* family is mediated by different signaling pathways.

### Pi Deficiency Enhances Plant Sensitivity to Ethylene

In addition to altering ethylene biosynthesis, Pi deficiency also alters plant sensitivity to ethylene. Pi deficiency-enhanced ethylene sensitivity has been reported for adventitious roots of maize seedlings (He et al., 1992), basal roots of common bean (Basu et al., 2007), and lateral roots of white clover (Dinh et al., 2012). The enhanced ethylene sensitivity is also reflected in the induction of gene expression by Pi deficiency (Lei et al., 2011b) as discussed in more detail later in this article. The enhanced ethylene sensitivity in Pi-starved plants is probably achieved through the alteration of the expression of the genes that encode molecular components involved in the ethylene signaling pathway. ERFs are a group of AP2 (APETALA2) domain-containing transcription factors that bind to ethylene-responsive elements present in the promoters of many ethylene-responsive genes. These transcription factors serve either as activators or repressors of ethylene-mediated transcription. Some transcriptomic analyses have shown that the expression of several *ERF* genes, including *ERF1*, *ERF2*, and *ERF5*, is altered in Pi-starved *Arabidopsis* roots (Wang et al., 2002; Misson et al., 2005; Thibaud et al., 2010; Chacón-López et al., 2011; Kang et al., 2014). In the *phr1phl1* double mutant, the expression of at least eight *AP2/ERF* genes is attenuated (Bustos et al., 2010). Another mechanism for enhancing ethylene sensitivity may involve a change of protein abundance of some key components in the ethylene signaling pathway. When plants were grown under salinity conditions, the accumulation of EIN3 protein was increased while that of EBFs, which mediate EIN3 protein degradation, was decreased (Peng et al., 2014). Whether this is also the case for Pi-deficient plants requires investigation.

### Ethylene and Root Responses to Pi Starvation

When plants are grown under Pi deficiency, their RSA undergoes a dramatic change, i.e., a remodeling. The Pi deficiency-induced remodeling of RSA includes a cessation of primary root growth and an enhanced production of root hairs and lateral roots (López-Bucio et al., 2003). For maize and some species in the *Proteaceae* and *Casuarinaceae* families, the remodeling of RSA involves a production of adventitious roots and cluster-roots (CRs; He et al., 1992; Wang et al., 2015). Such remodeling of RSA results in an increase in root surface area for Pi absorption. The remodeling starts with a reduction of cell elongation followed by the progressive loss of meristematic cells, i.e., “meristem exhaustion” (Sánchez-Calderón et al., 2005). At later stages, cell proliferation is arrested, and cell differentiation takes place at the former meristematic and elongation regions of the primary root. In some plant species, the angle between the basal root and primary root is increased, which enhances the capacity of roots to forage Pi in the top soil (Basu et al., 2007). The remodeling of RSA triggered by Pi deficiency is thought to be an active developmental response controlled by internal genetic programs because *Arabidopsis* mutants *lpi* and *lpr1* show normal growth of primary roots with an unexhausted meristem under Pi deficiency (Sánchez-Calderón et al., 2006; Svistoonoff et al., 2007).

Under normal growth conditions, application of ethylene (or ACC) to *Arabidopsis* plants inhibits primary root growth and enhances root hair production. This treatment results in meristem exhaustion of the primary root. *Arabidopsis* mutant that overproduces ethylene (*eto1*; Wang et al., 2004) or that has constitutive *ctr1* (Kieber et al., 1993) also exhibit reduced primary root growth and increased production of root hairs. These ethylene-induced root growth phenotypes mimic the plant root responses triggered by low Pi, suggesting that ethylene biosynthesis and signaling are involved in the Pi deficiency-triggered remodeling of RSA.

The roles of ethylene in Pi deficiency-induced remodeling of RSA have been investigated using both pharmacological and genetic approaches. Regarding the effect of ethylene on primary root growth under Pi deficiency, Chacón-López et al. (2011) found that inhibiting ethylene biosynthesis with AVG or ethylene perception with Ag^+^ restricted the low Pi-induced meristem exhaustion of the primary root. This suggested that ethylene is involved in the Pi deficiency-induced inhibition of primary root growth. Our laboratory also found that application of Ag^+^ reduces the inhibition of primary root growth triggered by Pi deficiency (Yu et al., 2012). The *Arabidopsis* mutant *hps4* (*hypersensitive to Pi starvation 4*) shows enhanced sensitivity to Pi deficiency in terms of Pi deficiency-induced inhibition of primary root growth and induction of APase activity. Under Pi sufficiency, the primary root of *hps4* was about 80% as long as WT (wild type) plants. Under Pi deficiency, the primary root growth was reduced for both the WT and *hps4*; however, this reduction was much greater for *hps4* than for the WT. *HPS4* encodes the SABRE protein. Although the precise biochemical function of SABRE is unknown, SABRE has been shown to antagonistically interact with ethylene to regulate root cell expansion (Aeschbacher et al., 1995). The hypersensitivity to Pi deficiency-induced inhibition of primary root growth is diminished when the *hps4* mutant is treated with Ag^+^ but not with AVG. Nagarajan et al. (2011) showed that overexpression of an *Arabidopsis* high-affinity Pi transporter, Pht1:5, reduced primary root growth and increased root hair production under both Pi sufficiency and deficiency. These phenotypes could be reversed by application of AVG or Ag^+^, indicating that ethylene biosynthesis had been altered in the *Pht1:5*-overexpressing lines. The *Pht1:5*-overexpressing lines also showed a disruption of the shoot to root Pi ratio, suggesting that an altered Pi homeostasis might enhance ethylene biosynthesis and/or signaling, which in turn, might modulate primary root growth. More experimental evidence is needed, however, to support this proposed link between altered Pi homeostasis and enhanced ethylene biosynthesis and/or signaling. Interestingly, when Ma et al. (2003) investigated the role of ethylene in Pi starvation-induced inhibition of primary root growth, they found that inhibition of ethylene production by AVG or inhibition of ethylene action by MCP increased primary root growth under high Pi but decreased primary root growth under low Pi. This result differs from the observations of López-Bucio et al. (2002) and Yu et al. (2012). The discrepancy could be due to the differences in experimental conditions used by the research groups. For example, the degree of low Pi stress in the different media differed, and whether the effect of ethylene on primary root growth is stimulatory or inhibitory could depend on a subtle difference in Pi concentrations, even though all concentrations were low. In fact, ethylene can be as both a promoter or an inhibitor for root growth depending on its concentration (Pierik et al., 2006). Thus, after inhibitors were applied in the experiments of Ma et al. (2003), the concentration of ethylene in Pi-deficient roots may have been far below the optimal level for sustained primary root growth under Pi deficiency.

In young maize seedlings, Pi deficiency induces the formation of aerenchyma (tissue with large cortical gas spaces) in their adventitious roots (He et al., 1992). When Ag^+^ or AVG was added to the nutrient solution, the formation of aerenchyma was blocked. Furthermore, when ethylene was added to the air of the growth chamber at a concentration as low as 1.0 μL/L, the aerenchyma formation was strongly promoted in Pi-starved roots relative to Pi-sufficient roots. Because the production of ethylene was decreased in Pi-starved maize seedlings in these experiments, it seemed that ethylene perception or sensitivity rather than ethylene production was involved in the formation of aerenchyma triggered by Pi deficiency. A similar case was found for tomato plants. Low Pi induced the formation of adventitious root in WT tomato plants but not in the ethylene-insensitive cultivar “Never-ripe” (Kim et al., 2008). Pi deficiency, however, reduced ethylene production in both tomato genotypes. This again indicated that it is ethylene perception rather than ethylene production that is involved in the response of roots to Pi availability.

The effects of ethylene on lateral root formation in Pi starved-plants have also been investigated. Pi deficiency stimulates the formation of lateral roots of white clover (Dinh et al., 2012). A low concentration of ACC had little effect on the development of lateral roots under Pi sufficiency but caused a super-stimulation of lateral roots under Pi deficiency. Unlike in white clover, Pi deficiency in common bean reduced lateral root number and did not inhibit primary root growth (Borch et al., 1999). This resulted in a reduction of lateral root density. AVG treatment increased lateral root density in Pi-deficient plants but reduced lateral root density in Pi-sufficient plants. These responses could be reversed by exogenous ethylene, suggesting an involvement of ethylene in modulating lateral root formation in common bean under Pi deficiency.

When grown under Pi deprivation, most members of the *Proteaceae* and *Casuarinaceae* form CR. The formation of CR densely covered with long root hairs dramatically increases the surface area for secretion of root exudates, including organic acids, protons, and APases. This root response helps plants mobilize Pi from organophosphates or Pi fixed with metals. Application of ethylene did not induce CR formation under Pi sufficiency; however, in Pi-deficient plants, the inhibition of ethylene production by Co^2+^ completely suppressed CR formation (Wang et al., 2015). Associated with this, the authors found a moderate increase in the expression of an *ACO* gene in the pre-emergent CR but a dramatic increase in *ACO* expression in the maturing CR.

In many soils, Pi availability is greatest in the upper layers and decreases with depth. Analyses of different genotypes of bean indicated that the degree of growth angle of basal roots was closely correlated with the extent of Pi acquisition of plants under low Pi availability (Basu et al., 2007). Ethylene sensitivity was higher for plants grown under Pi deficiency than under Pi sufficiency, and basal roots produced from the uppermost whorl were more sensitive to ethylene than those from the lower-most whorl. Thus, the growth angle of basal roots was strongly correlated with ethylene sensitivity but not with ethylene production.

The earliest visible change in the morphology of roots responding to Pi deficiency is the enhanced production of root hairs, including an increase in both root hair density and root hair length (Bates and Lynch, 1996). The increase in root hair length is due to an increase in both growth rate and growth duration. In *Arabidopsis*, root hairs are produced from H cells that are located over the intercellular space between two underlying adjacent cortical cells; however, not all H cells form root hairs. Under normal growth conditions, treatment with ACC or a mutation in the *CTR1* and *ETO1* genes causes a dramatic increase in root hair production that mimics the effect of Pi deficiency. Schmidt and Schikora (2001) found that low Pi could not fully restore the root hair density of *ein2* and *etr1* mutants to that of the WT. And, for the three ethylene biosynthesis inhibitors that they used (AVG, Co^2+^, and aminooxyacetic acid), only Co^2+^ significantly blocked the Pi deficiency-induced increase in the root hair density of the WT plants. Thus, the authors concluded that the canonical ethylene signaling pathway was not involved in the development of extra root hairs in response to Pi deficiency. However, the research of Zhang et al. (2003) strongly suggested that ethylene is involved in the Pi deficiency-enhanced production of root hairs. These authors found that under Pi deficiency, treatment with inhibitors of either ethylene biosynthesis or ethylene signaling significantly reduced root hair density and root hair length. Although all ethylene-insensitive mutants still responded to Pi deficiency with increased root hair density and length, the extent of the increase was much lower than that of the WT. These results indicated that ethylene is indeed involved in the enhanced production of root hairs induced by Pi deficiency.

Anatomic examination, however, revealed some similarities and differences in the effects of ethylene and Pi deficiency on root hair formation (Zhang et al., 2003). The similarity is that both low Pi and ethylene shortened the length of trichoblast cells and that AVG added to Pi-deficient plants increased the length of trichoblast cells. The differences include: (1) Low Pi increases the number of cortical cells, but ethylene does not; (2) Ethylene increase the percentage of H cells that form root hairs, but low Pi does not. These differences suggested that low Pi and ethylene may use the different gene activation mechanisms to regulate root hair formation. It will be interest to compare the transcriptomic changes in Pi-starved and ethylene-treated roots to identify the common and distinct targets of low Pi and ethylene. These information will help us further understand the molecular mechanisms of how ethylene mediates the root hair growth under Pi deficiency.

Further analysis demonstrated that there is an interaction between low Pi and ethylene in regulating both root hair length and root hair density (Zhang et al., 2003). The reduction in trichoblast length in *ein2* and *ein4* was stronger with low Pi than with high Pi, indicating that the degree to which ethylene affects extra root hair production depends on Pi availability. And, *ein2* and *ein4* mutants or the WT treated with AVG had greater reduction of H cells forming hairs under low Pi than under high Pi. In addition, under Pi deficiency, all ethylene-insensitive mutants clearly showed a reduction in root hair length, but the reduction varied for different mutants under high Pi. Cho and Cosgrove (2002) found that root hair elongation for ethylene-insensitive mutants or for plants treated with AVG was relatively normal under high Pi but was reduced under low Pi, providing additional evidence of an interaction between ethylene and Pi availability.

### Ethylene and Pi Transcriptional Regulation

In searching for molecular components involved in transcriptional responses of plants to Pi starvation, Lei et al. (2011b) performed a screen for *Arabidopsis* mutants with altered transcriptional response. They used a transgenic line that carries a LUC gene fused to the promoter of the high-affinity Pi transporter AtPT2. The transcription of *AtPT2* is induced by Pi starvation. Using this marker line, the authors identified the *Arabidopsis* mutant *hps2* (*hypersensitive to Pi starvation2*), which showed hyper-induction of the *AtPT2::LUC* gene by Pi deficiency. *hps2* is a new allele of the *CTR1* gene (**Figure 2B**). Furthermore, under Pi deficiency, treatment of *Arabidopsis* plants with Ag^+^ suppressed the induction of *AtPT2* whereas the addition of ACC dramatically enhanced its expression. Accordingly, the expression of *AtPT2* was partially blocked in *ein2* but was enhanced in *eto1*. A similar expression pattern was observed for several other PSI genes in the *hps2* and *ein2* mutants. These PSI genes included another high-affinity phosphate transporter, *AtPT1* (*Pht1; 1*; Muchhal et al., 1996); a non-coding transcript, *At4* (Burleigh and Harrison, 1999); an APase, *ACP5* (del Pozo et al., 1999); a ribonuclease, *RNS1* (Bariola et al., 1994; and *miR399d*, Fujii et al., 2005). The enhanced transcription of these PSI genes was also observed in the mutant *hps3*, which is another allele of *ETO1* (Wang et al., 2012), and in the mutant *hps4*, which has enhanced ethylene signaling (Yu et al., 2012). ETO1 protein is a member of the broad complex/tramtrack/bric-a-brac (BTB) protein superfamily that participates in substrate recognition during ubiquitin-mediated protein degradation (Christians et al., 2009). It directly binds to the C-terminal of ACS5 and mediates its degradation. When ETO1 is mutated, it causes an overproduction of ethylene in young seedlings (Wang et al., 2004). These results provided the first genetic evidence that ethylene signaling is involved in the transcriptional responses of plants to Pi deficiency. In another study using *M. falcata*, ACC induced the expression of the Pi transporter genes *MfPT1* and *MfPT5* under Pi-sufficient conditions, whereas both AVG and Co^2+^ blocked the low Pi-induced expression of these genes (Li et al., 2011). Taken together, these results indicate that ethylene positively regulates transcription of a subset of PSI genes. The incomplete blockage of PSI gene expression in the *ein2* mutant also indicates that ethylene is not the only mediator for the expression of these genes.

Interestingly, application of 25 μM ACC to young *Arabidopsis* seedlings under high Pi conditions barely induced the expression of *AtPT2*; under Pi deficiency, however, 0.5 μM ACC dramatically increased *AtPT2* expression (Lei et al., 2011b). These results suggested a synergistic interaction between low Pi and ethylene in mediating transcriptional Pi responses. This also provided another example of plant cells being sensitized to ethylene by low Pi.

### Ethylene and Induction of Acid Phosphatases

Induction and secretion of APases is a universal response of plants to Pi deficiency (Tran et al., 2010). The intracellular APases are believed to be involved in the remobilization of Pi from senescing tissues to young growing tissues whereas secreted APases are thought to be important for releasing Pi from organophosphates in the rhizosphere and thus increasing Pi availability for root uptake. The secreted APases are further classified into two groups: one group is released into the environment, and the other is tightly associated with the root surface after secretion. Members of the second group are called root-associated APases. Lei et al. (2011b) found that *hps2* also had enhanced APase activity on the root surface. In contrast, *ein2* exhibited reduced root-associated APase activity under Pi deficiency, indicating that ethylene is a positive regulator of PSI APase activity. The role of ethylene in regulating APase activity was further confirmed by the analyses of mutants *hps3* and *hps4* (Wang et al., 2012; Yu et al., 2012). Treatment with Ag^+^ suppressed the enhanced APase activity in *hps3* and *hps4*. Similarly, Li et al. (2011) showed that the induction of root APase activity in *M. falcata* was stimulated by ACC under Pi sufficiency but was blocked by AVG under Pi deficiency.

In *Arabidopsis*, AtPAP10 is a major PSI APase that is predominantly associated with the root surface after secretion (Wang et al., 2011, 2014a). Zhang et al. (2014) investigated how ethylene affects root-associated AtPAP10 activity at different regulatory steps. The transcription of *AtPAP10* was previously found to be increased by Pi starvation in the whole seedlings of *hps3* and *hps4* (Wang et al., 2012; Yu et al., 2012). The total root intracellular APase activity in *hps3* and *hps4*, however, did not significantly differ from that of the WT. When the transcription of *AtPAP10* was further analyzed using separated root and shoot tissues, Zhang et al. (2014) found that the transcription of *AtPAP10* did not significantly increase in ACC-treated seedlings or the *ctr1* mutant under Pi deficiency, nor did the accumulation of AtPAP10 proteins. Taken together, these results indicated that, in roots, ethylene mainly modulated the secretion of AtPAP10 protein or its enzymatic activity on the root surface. Some reports have shown that ethylene can increase H^+^-ATPase activity by up-regulating the expression of H^+^-ATPase genes (Waters et al., 2007; Wang et al., 2009; Staal et al., 2011), which may decrease the cytosolic pH or the pH on the root surface. In contrast, application of MCP blocked the change of cytosolic pH (Sundaresan et al., 2015). In humans and animals, cytosolic pH levels affect protein secretion (Paroutis et al., 2004). In addition, APase activity is sensitive to the change of pH. Thus, it is possible that ethylene may increase secretion of AtPAP10 proteins and stabilize AtPAP10 enzymatic activity on the root surface by modulating the cytosolic and root surface pH. More experimental evidence, however, is required to support this hypothesis.

### Ethylene and Anthocyanin Accumulation

Accumulation of anthocyanin is another hallmark response of plants to Pi starvation. The accumulation of anthocyanin is lower in *hps2*, *hps3*, and *hps4* mutants than in the WT under low Pi conditions (Lei et al., 2011b; Wang et al., 2012; Yu et al., 2012). In contrast, the Pi-starved *ein2* mutant shows increased anthocyanin content. Ag^+^-treated *Arabidopsis* seedlings also displayed increased accumulation of anthocyanin under Pi deficiency. Furthermore, Lei et al. (2011b) showed that the expression of four genes that encode three enzymes involved in anthocyanin biosynthetic pathway and one transcription factor regulating anthocyanin biosynthesis was increased in *ein2* but reduced in *ctr1*. These results demonstrate that ethylene is a negative regulator of Pi starvation-induced anthocyanin accumulation and that this regulation is achieved at least partly through the regulation of the transcription of the genes involved in the biosynthesis of anthocyanin.

### Ethylene’s Role in Local Signaling

Plant responses to Pi deficiency are controlled by a complex regulatory network involving both local and systemic signaling. Whether a Pi response is controlled by local or systemic signaling can be determined by examining whether the degree of the response depends on the local Pi level or the Pi status of the whole plant. Using this approach, Ticconi et al. (2004) found that the remodeling of RSA triggered by Pi deficiency was regulated by the local, external Pi level. They first germinated *Arabidopsis* seeds on a Pi-sufficient medium for 5 days and then transferred the seedlings to an agar plate that contained a Pi-sufficient medium in the upper half and a Pi-deficient medium in the lower half, or *vice versa*. After the roots had grown for another 6 days, lateral root production had increased in the parts of the root that contacted the Pi-deficient medium but decreased in the parts that contacted the Pi-sufficient medium. The *Arabidopsis phf1* mutant has defects in the ER exit of high-affinity Pi transporters, which greatly impairs Pi uptake by roots (González et al., 2005). When these plants were grown on a Pi-sufficient medium, their internal Pi level was only 20% of the WT, but no remodeling of RSA was observed (Thibaud et al., 2010). Furthermore, Thibaud et al. (2010) found that injection of a high concentration of Pi into the shoots of *Arabidopsis* plants grown on a Pi-deficient medium could not suppress the Pi deficiency-induced remodeling of RSA. Together, these results demonstrated that it is the local, external Pi level rather than the internal Pi status of the whole plant that regulates the remodeling of RSA. The requirement for ethylene in the Pi deficiency-induced remodeling of RSA indicates that ethylene is involved in local Pi signaling.

The *Arabidopsis* mutant defective in LPR1 and its close paralog LPR2 are insensitive to the Pi deficiency-induced inhibition of the primary root growth (Svistoonoff et al., 2007). In contrast, the *Arabidopsis* mutant with functional disruption of PDR2, which encodes a P5-type ATPase, exhibits an exaggerated short-root phenotype under Pi deficiency owing to meristem exhaustion (Ticconi et al., 2004, 2009). SCR and SHR are two key regulators of root patterning. PDR2 is required for maintaining the levels of SCR protein and SHR trafficking from stele into endodermis. Based on genetic analysis, PDR2 was proposed to act upstream of LPR1/LPR2 to adjust meristem activity in an ER-resident pathway. The roots of WT plants accumulated more Fe under Pi deficiency than under Pi sufficiency (Svistoonoff et al., 2007; Ward et al., 2008; Zheng et al., 2009; Lei et al., 2011a). On a Fe-free medium, the inhibition of primary root growth was abolished. Thus, Ward et al. (2008) hypothesized that Pi deficiency triggers the inhibition of primary root growth by enhancing the accumulation of Fe in the root meristem, which results in severe damage to root cells. In a recent study, Müller et al. (2015) demonstrated that LPR1 is a ferroxidase. The root meristem of *lpr1* contains reduced levels of Fe^3+^ under Pi deficiency, which makes the root tip growth insensitive to inhibition caused by Pi deficiency. In contrast, *pdr2* accumulates increased levels of Fe^3+^, which generates high levels of reactive oxygen species (ROS). The high level of ROS, in turn, causes the increased deposition of callose which impairs the trafficking of SHR, thus restricting root tip growth. Ethylene positively regulates Fe homeostasis in plants by up-regulating the expression of the genes involved in Fe acquisition (Waters et al., 2007; García et al., 2011). Thus, it is also possible that ethylene mediates Pi deficiency-induced inhibition of primary root growth by enhancing Fe accumulation in root tips.

As previously mentioned, AtPAP10 is a PSI APase that is predominantly associated with the root surface after secretion. Using split-root experiments, Zhang et al. (2014) demonstrated that although the transcription of *AtPAP10* is systemically controlled (i.e., affected by the Pi status of whole plant), AtPAP10 protein accumulation and enzymatic activity on the root surface are regulated by local Pi levels. Once the mRNA of *AtPAP10* is produced, the subsequent accumulation and secretion of AtPAP10 protein and perhaps also the protein’s enzymatic activity on the root surface are controlled only by local signaling. Because ethylene mainly participates in the secretion of AtPAP10 proteins or stabilization of AtPAP10 enzymatic activity on the root surface during the induction of root-associated AtPAP10 activity, ethylene could be regarded as a local signal in regulating the induction of AtPAP10 activity. This represents another example that ethylene functions in local signaling besides being involved in the control of remodeling of RSA.

Although it is now evident that ethylene is involved in local Pi sensing and signaling, the mechanism by which ethylene regulates local signaling is largely unknown. Thibaud et al. (2010) dissected the transcriptional responses controlled by local and systemic signaling. They found that the transcription of the genes involved in development, stress responses, and hormonal signaling is controlled by local signaling. The locally regulated genes include *ERF1* and *ERF2*. These results provide further support for the role of ethylene in local Pi signaling. The next important task will be to identify the direct downstream genes that are targets of ethylene and that participate in local Pi signaling.

### Ethylene’s Role in Systemic Signaling

**FIGURE 3:**
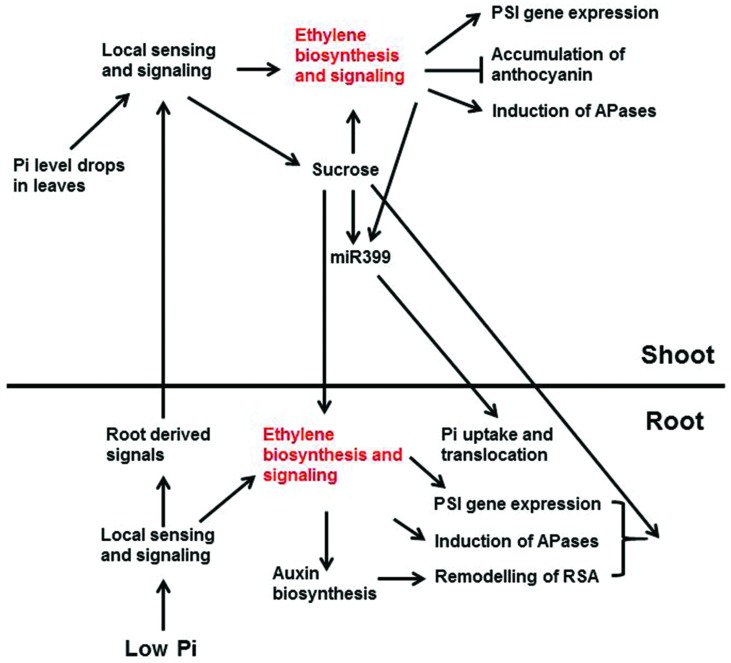
**A schematic model showing the interactions between ethylene and other Pi signaling components and Pi deficiency responses that these interactions are involved.** Low Pi is perceived by unidentified intracellular and/or extracellular sensors in root, which activates local signaling pathways and the synthesis of systemic signals. The activated local signaling pathways then trigger the expression of PSI genes, the induction of APases, and the remodeling of RSA. These response are egulated partly through ethylene biosynthesis and signaling. The ethylene-mediated remodeling of RSA may also be achieved by enhancing auxin biosynthesis and signaling. The root-derived systemic signals are translocated to shoot via xylem. These signals, together with the drop of Pi level in shoots, elicit the local sensing and signaling there. The local signaling in shoots increases ethylene biosynthesis and signaling, as well as the synthesis of systemic signals, such as sucrose. The enhanced ethylene biosynthesis and signaling induce the expression of PSI genes and APase activity, but suppress the accumulation of anthocyanin. The increased sucrose level, in turn, can reinforce the ethylene biosynthesis and signaling and induces the production of miR399. The induction of miR399 is also modulated by ethylene and transcription factor PHR1 (not show in the figure). The shoot-derived sucrose and miR399 then move down to roots through phloem transport. In roots, sucrose is critical for the expression of some PSI genes and the induction of APase activity, perhaps also the remodeling of RSA (not discussed in this article), and the accumulation of miR399 enhances Pi uptake and translocation to shoots. Arrows indicate promotion, and the perpendicular line indicate inhibition (Lei et al., 2011b).

Researchers have hypothesized that when the external Pi level drops, the root tissues sense the change in Pi availability and send warning signals through the xylem to the shoots (Chiou and Lin, 2011; **Figure 3**). Once these signals arrive in shoots, they trigger Pi-deficiency responses in the shoots including enhanced PSI gene expression, reduced photosynthetic activity, inhibition of shoot growth, increased anthocyanin accumulation, and induction of intracellular APase activity. At the same time, the shoots send signals to the roots through the phloem to regulate Pi responses in roots including enhanced high-affinity Pi transporter activity and induction of APase activity. Cytokinins, strigolactones, and Pi itself have been proposed to be root-to-shoot signals, whereas sucrose and miRNA399 are believed to be the shoot-to-root signals (Lin et al., 2014). Enhanced ethylene biosynthesis and expression of ethylene biosynthetic genes in shoots have been observed under Pi deficiency (Misson et al., 2005; Kim et al., 2008), suggesting that ethylene is involved in the Pi responses in shoots. Whether the increase of ethylene biosynthesis in shoots is triggered by local sensing due to the drop of Pi levels in the shoots or by the signals from the roots, however, is not known.

Thibaud et al. (2010) showed that the transcription of PSI genes that participate in Pi transport, signaling, and recycling is regulated by systemic signaling. The PSI genes whose expression is regulated by ethylene also belong to these functional categories (Lei et al., 2011b; Wang et al., 2012; Yu et al., 2012). The transcription of the Pi transporters Pht1;1 and Pht1;4, the APase ACP5, the RNase RNS1, and the Pi signaling molecules miRNA399, At4, and IPS1 is enhanced in *hps2*, *hps3*, and *hps4* but reduced in *ein2* or in the plants treated with Ag^+^. Thibaud et al. (2010) further demonstrated that the promoters of most systemically regulated PSI genes contain the P1BS element, which is the binding site of the PHR1 transcription factor, indicating that PHR1 is an important component of the systemic signaling pathway. The reduced expression of one *ACS* gene and at least eight *AP2/ERF* genes in the *phr1phl1* mutant suggests that ethylene biosynthesis and signaling themselves are also controlled by systemic signaling. Thus, ethylene might be part of a regulatory loop involved in the systemic control of PSI gene expression. Once activated, however, how ethylene signaling functions in systemic control of PSI gene expression is unknown.

In the *phr1* mutant, anthocyanin accumulation is greatly attenuated, indicating that this response to low Pi is systemically controlled (Rubio et al., 2001). As discussed before, the accumulation of anthocyanin is enhanced in *ctr1* and *eto1* but reduced in *ein2*, indicating that ethylene is a negative regulator of this systemic response (Lei et al., 2011b; Wang et al., 2012). These results reveal another function of ethylene in the systemic control of plant responses to Pi deprivation.

## Interaction between Ethylene and Other Signals

The past studies have indicated that the regulation of plant responses to Pi deficiency is complex and involves crosstalk among different signaling pathways. The precise control and coordination of these multi-faceted responses undoubtedly depends on an efficient interaction among the different signals. Here, we discuss how ethylene interacts with sucrose, auxin, and miRNA399 to regulate Pi-deficiency responses.

### Ethylene and Sucrose

Growing evidence indicates that sucrose is a key systemic signal that globally regulates Pi-starvation responses. In several plant species, sucrose biosynthesis increases under low Pi availability (Hammond and White, 2008). Also, the expression of the genes involved in the synthesis, translocation, and degradation of sucrose is altered when plants are grown under Pi deficiency (Hammond et al., 2003; Wu et al., 2003; Misson et al., 2005; Muller et al., 2007). In addition to functioning as a carbon source, the sucrose delivered to the roots from leaves acts as a signal to initiate changes in gene expression, metabolism, and development in roots (Hammond and White, 2011). Karthikeyan et al. (2007) found that the level of PSI gene expression was positively correlated with the concentrations of sucrose in the growth medium under Pi deficiency. When plants were grown in the dark, the expression of PSI genes was greatly reduced; this reduction in PSI gene expression, however, was prevented by adding sucrose to the growth medium. Similarly, Liu et al. (2005, 2010) observed that application of sucrose stimulated accumulation of *LaPT1* (a Pi transporter), *LaSAP1* (an APase) and miR399 transcripts in dark-grown white lupin and common bean under Pi sufficiency. Furthermore, the use of stem-girdling to disrupt the phloem transport of photosynthates to P-deficient roots resulted in the suppression of the expression of these genes. Jain et al. (2007) reported that sucrose is required for Pi deficiency-induced lateral root proliferation and root hair formation. In addition, exogenous application of sucrose, like Pi deficiency, induced CR formation and PSI gene expression in Pi-sufficient white lupin (Zhou et al., 2008). Definitive evidence for the role of sucrose in plant responses to Pi starvation came from the study of the *Arabidopsis* mutant *hps1* (Lei et al., 2011a). *hps1* overexpresses the *SUC2* gene due to a T-DNA insertion in the promoter of the *SUC2* gene. SUC2 is the only transporter involved in the phloem loading of sucrose in mesophyll cells. *hps1* accumulates a high level of sucrose in both roots and shoots and is hypersensitive in almost all aspects of plant response to Pi starvation. Lei et al. (2011a) further showed that the *suc2-5* mutant had enhanced expression of the Pht1:4 Pi transporter in shoots but reduced expression in roots, as well as reduced root-associated APase activity. These results were consistent with the high accumulation of sucrose in shoots and low level of sucrose in roots in this mutant. The reduced root-associated APase activity was also observed in another mutant allele of *SUC2*, *pho3* (Zakhleniuk et al., 2001; Lloyd and Zakhleniuk, 2004).

In several plant species, sucrose increases ethylene production in a sucrose concentration-dependent manner (Philosoph-Hadas et al., 1985; Kobayashi and Saka, 2000; Jeong et al., 2010; **Figure 3**). The sucrose-induced formation of CR in white lupin was completely suppressed by application of Co^2+^ (Wang et al., 2015). In addition, Co^2+^ also suppressed Pi deficient-induced CR formation. Together, these results suggest that sucrose induces CR formation via the induction of ethylene biosynthesis (**Figure 3**).

Both sucrose and ethylene are positive regulators for the induction of AtPAP10 APase activity on the root surface (Lei et al., 2011a,b; Wang et al., 2012; Yu et al., 2012). Zhang et al. (2014) further investigated the relationship between sucrose and ethylene in the regulation of AtPAP10 activity. Under Pi deficiency, *hps1* had enhanced APase activity while *ein2* had reduced APase activity on the root surface. The double mutant *hps1ein2* displayed root-associated AtPAP10 activity that was intermediate between that of *hps1* and *ein2*. Also, when treated with Ag^+^, the AtPAP10 activity in WT plants was partially reduced. When *ctr1* was grown in the dark on a sucrose-free Pi-deficient medium, however, the induction of the AtPAP10 activity on the root surface was completely blocked, although ethylene signaling was constitutively activated in the mutant. Thus, ethylene’s induction of AtPAP10 activity depends on sucrose, but sucrose’s function does not depend on ethylene. Further study indicated that sucrose was largely required for the induction of *AtPAP10* transcription while ethylene only modulated the secretion of AtPAP10 protein or AtPAP10 enzymatic activity on the root surface (Zhang et al., 2014). This is understandable because if *AtPAP10* mRNA is not transcribed due to the absence of sucrose, ethylene signaling, even if is constitutively activated, will not increase the AtPAP10 activity on the root surface. In addition, the above results also indicate that ethylene is not the only component that controls the induction of APase activity; even if the ethylene pathway is completely blocked by the *ein2* mutation or by treatment with Ag^+^, the induction of APase is only partially abolished.

### Ethylene and Auxin

Auxin also plays an important role in controlling Pi deficiency-induced remodeling of RSA (López-Bucio et al., 2002; Nacry et al., 2005; Jain et al., 2007; Pérez-Torres et al., 2008). López-Bucio et al. (2002) showed that Pi-deprived plants were more sensitive to exogenously applied auxin than Pi-replete plants with respect to the arrest of primary root growth and enhanced formation of lateral roots. This enhanced auxin sensitivity was found to result from the enhanced expression of the auxin receptor TIR1 (Pérez-Torres et al., 2008). The enhanced TIR expression accelerates the degradation of AUX/IAA (AUXIN/INDOLE-3-ACETIC ACID) auxin response repressors, thus releasing repression of transcription factor ARF19 that is involved in the formation of lateral roots. Nacry et al. (2005) proposed that Pi deficiency causes: (1) an over-accumulation of auxin in the root apex of primary root and young lateral roots; (2) an over-accumulation of auxin or an increased auxin sensitivity in the lateral primordia; (3) a decrease in auxin concentration in the lateral primordia initiation zone of the primary roots and in old laterals. Using auxin transport inhibitor or the mutants with defects in auxin transport, the authors also showed that the changes in local auxin concentrations was achieved through the changes in auxin transport rather than auxin biosynthesis. Given that both ethylene and auxin are involved in the Pi deficiency-induced remodeling of RSA, ethylene may cooperate with auxin to regulate root growth. In fact, several lines of evidence have indicated that ethylene promotes auxin biosynthesis and transport to modulate root development (Osmont et al., 2007; Růzicka et al., 2007; Swarup et al., 2007; Stepanova and Alonso, 2009).

The *Arabidopsis* mutant *hps4* is hypersensitive to the Pi deficiency-induced inhibition of primary root growth (Yu et al., 2012). In this mutant, the expression of several genes related to auxin biosynthesis is increased. In addition, the *hps4* root tip produced twice as much auxin as that of the WT under Pi deficiency. The hypersensitivity of *hps4* to Pi deficiency was suppressed when the plants were treated with Ag^+^ but not with AVG. These results suggested that the enhanced ethylene signaling in *hps4* might increase auxin biosynthesis in the root tip, thus enhancing the inhibition of the primary root growth (**Figure 3**). In white clover, Pi deficiency increases the ethylene sensitivity in roots (Dinh et al., 2012). ACC treatment induced a high expression of the auxin-responsive DR5::GUS marker gene in the root apex. To separate the effect of ACC on auxin biosynthesis from auxin transport, the authors applied auxin transport inhibitor to Pi deficient-roots. The results showed that the ACC-enhanced DR5::GUS expression could not be suppressed by the auxin transport inhibitor, suggesting that the effect of ACC is on auxin biosynthesis, although the role of ACC on auxin transport cannot be strictly excluded. This result is also consistent with what observed by Yu et al. (2012).

### Ethylene and miR399

The first and also the best characterized miRNA involved in Pi-deficiency response is miR399. The expression of miR399 is highly induced in both shoots and roots by a decrease in external Pi or copper levels, but is reduced by iron deficiency (Fujii et al., 2005; Chiou et al., 2006; Buhtz et al., 2010). A detailed time course study indicated that the increase of miR399 in the shoot occurs prior to that in the root (Lin et al., 2008). Reciprocal grafting experiments further showed that miR399 could move from shoot to root (Lin et al., 2008; Pant et al., 2008). Observation of the increased accumulation of miR399 in the phloem sap in Pi starved-*Arabidopsis* and *Brassica* plants (Buhtz et al., 2008; Pant et al., 2008) also supported that miR399 is synthesized in shoots and is translocated to roots in order to act as a systemic signal that regulates Pi uptake. miR399 enhances Pi acquisition by direct cleavage of the mRNA that encodes the ubiquitin E2 conjugase PHO2, which is involved in the ubiquitin-mediated protein degradation pathway (Aung et al., 2006; Bari et al., 2006; Chiou et al., 2006). Overexpression of *miRNA399* or functional disruption of PHO2 leads to the over-accumulation of Pi in shoots. By screening for *pho2* suppressors and using quantitative membrane proteomics, Liu et al. (2012) and Huang et al. (2013) identified PHO1 and a group of high-affinity Pi transporters (PHT1) as the substrates of PHO2. PHO1 and PHT1 transporters are responsible for the translocation of Pi from roots to shoots and for the uptake of Pi from the external environment. When plants are exposed to Pi deficiency, miR399 is rapidly induced and degrades the mRNA of *PHO2*. The down-regulation of *PHO2* expression increases the stability of PHO1 and PHT1 proteins, thus enhancing Pi uptake in roots and Pi translocation from roots to shoots.

Transcription of the precursor of miR399d is enhanced in the *Arabidopsis* mutants *hps2*, *hps3*, and *hps4*, but the expression of miR399d is reduced in *ein2* (Lei et al., 2011b; Wang et al., 2012; Yu et al., 2012). These results demonstrated that ethylene positively regulates the expression of miR399d. Interestingly, Liu et al. (2010) found that the induction of miR399 was completely blocked in dark-grown or stem-girdled white lupin, suggesting that sucrose is required for miR399 expression (**Figure 3**). Given that an increase in sucrose level can induce ethylene biosynthesis, ethylene might act downstream of sucrose to affect the expression of miR399. Also, because the expression of miR399 is completely abolished in the absence of sucrose but is only partially blocked in the *ein2* mutant, it seems that ethylene functions in a branch of the pathway downstream of sucrose to modulate the expression of miR399 and thus to fine-tune Pi uptake by roots.

## Conclusion and Perspectives

Based on genetic, pharmacological, biochemical, and physiological data, it is now evident that ethylene plays an important role in mediating plant responses to Pi deficiency. Pi deficiency increases ethylene biosynthesis and signaling in both roots and leaves. Early studies showed that ethylene is involved in Pi deficiency-induced inhibition of primary root growth and enhanced production of root hairs. Recent research indicates that ethylene also regulates expression of PSI genes, induction of APases, and accumulation of anthocyanin under Pi deficiency. Ethylene participates in both transcriptional and post-transcriptional regulation of plant Pi responses. Moreover, ethylene interacts with other signals, such as sucrose, auxin, and miRNA399, to regulate both local and systemic signaling. Also, it should be noted that although ethylene clearly plays an important role in mediating multiple plant responses to Pi deficiency, there is no single Pi response that is completely under the control of ethylene.

Although the role of ethylene in mediating plant responses to Pi deficiency is well established, the following important questions remain regarding the underlying mechanisms: (1) How do plants sense the change in Pi availability to elicit the production of ethylene or to enhance ethylene sensitivity? (2) Which specific cells are the targets for ethylene action? (3) Through which downstream components does ethylene mediate plant responses to Pi starvation? To answer these questions, we need to identify the transcription factors that bind to the promoters of ethylene biosynthetic genes, such as *ACS* and *ACO*, and to understand how the expression of these transcription factors is regulated by Pi deficiency. The expression of *ACS* and *ACO* genes and the stability of these proteins are also regulated by CDPK and MAPK signaling pathways (Kim et al., 2003; Hernández Sebastià et al., 2004; Liu and Zhang, 2004). A recent report indicated that *Arabidopsis* MKK9-MPK3/MPK6 pathway is involved in the maintenance of Pi homeostasis by regulating transcription of Pi acquisition-related genes (Lei et al., 2014). It is not known, however, whether this pathway regulates Pi homeostasis by affecting the expression or activity of ACS and ACO. Thus, it may also worth to investigate the molecular link between this MAPK pathway and ethylene biosynthesis/signaling under Pi deficiency.

To identify the cells that are the targets for ethylene effects, researchers might use a cell type-specific promoter to drive a mutated ethylene receptor gene *etr1* to specifically block the action of ethylene in those cells; the change in Pi response would then indicate whether those cells are involved. This approach has been successfully used to dissect the role of ABA signaling in mediating the response of root growth to drought stress (Duan et al., 2013). To identify the downstream targets of ethylene signaling that are directly involved in plant Pi responses, researchers can combine genomic and genetic approaches. For example, EIN3 and EIL1 are two key transcription factors that regulate a suite of downstream ethylene-responsive genes. A transcriptomic analysis would indicate those genes whose expression is blocked in the Pi deficient-*ein3eil1* mutant. Using such an approach, researchers could identify a battery of genes that directly participate in plant responses to Pi deficiency, and these might include, for example, the genes of cell wall proteins that are directly involved in the elongation of root hairs. This approach could also reveal the gene regulatory network that ethylene uses to control specific Pi responses. In addition, by using a Pi starvation-responsive marker line (such as the plant carrying the *AtPT2::LUC* marker gene), researchers could screen for a mutant that uncouples the interaction between ethylene and Pi; thus identifying the molecular components involved in the regulation of ethylene sensitivity under Pi deficiency. With increased knowledge in these areas, we will better understand how ethylene functions in plant responses to Pi deficiency and how overall plant responses to Pi deficiency are regulated at the molecular level.

## Author Contributions

LS drafted the manuscript. LS and DL revised the manuscript.

## Conflict of Interest Statement

The authors declare that the research was conducted in the absence of any commercial or financial relationships that could be construed as a potential conflict of interest.

## Abbreviations

ACC: 1-aminocyclopropane-1-carboxylate
ACO: ACC OXIDASE
ACP5: ACID PHOSPHATASE TYPE 5
ACS: ACC SYNTHASE
AdoMet: *S*-adenosyl methionine
APase: acid phosphatase
AP2/ERF: APETALA2/ETHYLENE RESPONSE FACTOR
At4: *Arabidopsis thaliana* cDNA 4
ARF19: AUXIN RESPONSE FACTOR 19
AVG: aminoethoxyvinyl glycine
CDPK: CALCIUM-DEPENDENT PROTEIN KINASE
CHIB: CHITINASE B
CR: cluster root
CTR1: CONSTITUTIVE TRIPLE RESPONSE 1
EBF: EIN3-BINDING F-BOX
EIL1: EIN3-LIKE 1
EIN2: ETHYLENE INSENSITIVE 2
EIN3: ETHYLENE INSENSITIVE 3
EIN4: ETHYLENE INSENSITIVE 4
FLS2: FLAGELLIN SENSITIVE 2
ER: endoplasmic reticulum
ERS1: ETHYLENE RESPONSE SENSOR 1
ETR1: ETHYLENE RESPONSE 1
ETR1: ETHYLENE RESPONSE 1
ETO1: ETHYLENE OVERPRODUCTION 1
hps: hypersensitive to phosphate starvation
IPS1: INDUCED BY PHOSPHATE STARVATION 1
LPR1: LOW PHOSPHATE ROOT 1
LUC: luciferase
MAPK: MITOGEN-ACTIVATED PROTEIN KINASE
MCP: 1-methylcyclopropene
PAP10: PURPLE ACID PHOSPHATASE 10
PDR2: PHOSPHATE DEFICIENCY RESPONSE 2
PHL1: PHR1-LIKE 1
PHO2: PHOSPHATE 2
PHR1: PHOSPHATE STARVATION RESPONSE 1
Pht1(AtPT1): PHOSPHATE TRANSPORTER 1
Pi: phosphate
PORA: PROTOCHLOROPHYLLIDE OXIDOREDUCTASE A
PSI: phosphate starvation induced
RNS1: RIBONUCLEASE 1
RSA: root system architecture
SCR: SCARECROW
SHR: SHORT-ROOT
SUC2: SUCROSE TRANSPORTER 2
TIR1: TRANSPORT INHIBITOR RESPONSE 1.
